# Chronic cough conundrum: a case report of a new diagnosis of HIV and pulmonary Kaposi’s sarcoma

**DOI:** 10.1186/s12890-017-0395-5

**Published:** 2017-03-20

**Authors:** Pamela P. Bailey, Marylou M. Dryer, John P. Piper, Sajjad Ahmad

**Affiliations:** 1 4755 Ogletown-Stanton Road, Suite 2E70, Newark, DE 19718 USA; 2 Omega Professional Center, C-78-80 Omega Drive, Newark, DE 19713 USA; 3 4745 Ogletown-Stanton Road, MAP 1, Suite 220, Newark, DE 19713 USA

**Keywords:** Kaposi’s sarcoma, HIV, Cough

## Abstract

**Background:**

When evaluating a common complaint such as cough, clinicians should rely on a patient’s history and physical to guide them, but also not diverge from guidelines in screening and testing lest certain diagnoses be overlooked.

**Case Presentation:**

A 44 year old Hispanic male presented to a pulmonologist’s office after failing multiple courses of antibiotics for chronic cough, now six months in duration. He described intermittent scant hemoptysis and an evanescent migratory non-pruritic rash occasionally noted on his trunk or limbs. Due to financial concerns, the patient initially agreed only to limited testing. Eventually bronchoscopy was pursued, but results were pending when the patient presented to the emergency room with worsening dyspnea, blood-tinged sputum and weight loss. A diagnosis of Kaposi’s sarcoma (KS) of the lung was confirmed by histopathologic staining and HIV/AIDS was confirmed (HIV1 PCR 70,900 copies/mL, CD4 count 26 cells/mm^3^). He had repeatedly denied HIV risk factors to all providers, but once the diagnosis was established, he confirmed sexual promiscuity prior to his marriage greater than 10 years ago. He was started on HAART before initiating therapy for his KS due to concern for immune reconstitution syndrome worsening his pulmonary status.

**Conclusion:**

Pulmonary Kaposi’s sarcoma is an infrequent diagnosis, yet risk is significantly greater for those with HIV infection. Diagnosis is difficult, with both symptoms and radiographic findings being nonspecific and not distinctly different from the appearance of pulmonary opportunistic infections. Without treatment, patients with pulmonary KS have median survival of months, but with chemotherapy and HAART they may achieve relief from symptoms and improve survival.

Following recommended screening guidelines and furthering diagnostic evaluation for persistently symptomatic patients are key to uncovering potentially fatal disease even for patients whose symptoms may seem as common and benign as an irritating cough.

## Background

Cough is an incredibly common complaint. Clinicians should rely on a patient’s history and physical to guide them, but also not diverge from guidelines in screening and testing lest certain tests be overlooked which may reveal a rare cause.

## Case Presentation

**Fig. 1:**
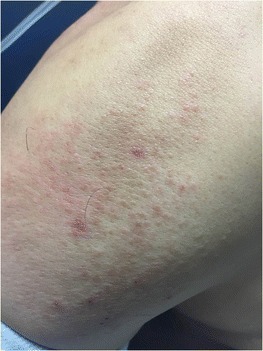
Evanescent migratory non-pruritic rash in patient found to have new diagnosis of HIV/AIDS and Kaposi’s Sarcoma

**Fig. 2:**
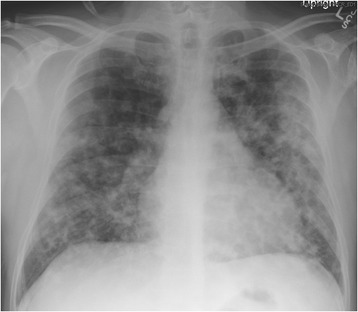
Upright AP Chest x-ray with bilateral, diffuse, airspace opacities was read as consistent with multilobar pneumonia possibly due to TB, MAI, aspergillosis or recurrent aspiration. The patient was ultimately determined to have pulmonary Kaposi’s sarcoma

A 44 year old Hispanic male was referred to a pulmonologist’s office after failing multiple courses of antibiotics for chronic cough, now 6 months in duration. He described intermittent scant hemoptysis and an evanescent migratory rash that was non-pruritic occasionally noted on his trunk or limbs (Fig. 1). His physical exam was otherwise unremarkable, including no adventitious lung sounds. Due to financial concerns, the patient initially agreed only to basic laboratory testing and imaging which was nonspecific (Fig. 2). His initial white blood cell count was 4.7 cells/nL, hemoglobin 13.9 grams/dL, platelet 209 cells/nL; his initial chest x-ray showed bilateral hazy nodular opacities. Eventually bronchoscopy was pursued, which was also unrevealing. No lesions were appreciated throughout the examined upper airway and lungs. Pathologic results and cultures were still pending when the patient presented to the emergency room with worsening dyspnea, blood-tinged sputum and weight loss. A diagnosis of Kaposi’s sarcoma (KS) of the lung was confirmed by histopathologic staining and HIV/AIDS was confirmed (HIV1 PCR 70,900 copies per mL, CD4 count 26 cells/mm^3^). He had repeatedly denied HIV risk factors to all providers, but once the diagnosis was confirmed, he reported sexual promiscuity prior to his marriage greater than 10 years ago. He was started on HAART before initiating therapy for his KS due to concern for immune reconstitution syndrome worsening his pulmonary status. He subsequently initiated chemotherapy for his KS and was maintaining overall good health.

## Discussion

Kaposi’s sarcoma is an uncommon diagnosis, yet risk of KS is 10,000 times greater among those with HIV infection [1]. In the United States between 1980 and 2007, there were an estimated 83,252 cases of KS for which AIDS was a contributing factor in 82% of cases. Incidence of KS peaked between 1990 and 1995 with subsequent decline after the introduction of HAART in 1996 [2, 3] since HHV8 (human herpesvirus 8, or Kaposi’s sarcoma associated-herpes virus) which causes KS can be better controlled with a stronger immune system.

Especially in the absence of characteristic skin and mucosal findings, diagnosis of KS may be hindered by nonspecific symptoms and radiographic findings making it difficult to distinguish from opportunistic infections. The disease is commonly associated with a violaceous or erythematous eruption that is generally papular or plaque-like on the skin and mucous membranes as originally described by the dermatologist after which Kaposi’s sarcoma is named [4]. In retrospect, presence of a rash in our patient is suspicious for possible association to his KS diagnosis, but was not classical in appearance and was not later able to be biopsied since it was transient. His rash is more likely to have been directly related to his HIV disease as an idiopathic pruritus has been reported with higher prevalence in patients with viral loads higher than 55,000 copies/mL [5].

Because pulmonary KS tends to progress from the subepithelial layer to invade the mucosal surfaces, bronchoscopic inspection is considered to be a highly sensitive method for diagnosis [6, 7]. As an example, in Pozniak’s cohort in Africa in 1992, only 1 patient of 47 with confirmed KS has a normal bronchoscopy; the others had the expected reddish, flat nodules [8]. The traditional endobronchial lesions in KS are maculopapular, reddish-purple in color, concentrated at airway bifurcations and parallel to the tracheal rings though can be seen throughout the tracheobronchial tree [8, 9]. These are vascular lesions making it difficult to weigh the risks and benefits of performing a biopsy which carries an approximate 30% risk for clinically significant hemorrhage [6, 7]. Additionally, the ability bronchoscopic biopsies to impact diagnosis is relatively low because of patchy submucosal involvement with diagnostic yield ranging from 7 to 60% [6, 8–10]. Once again, despite abnormal bronchoscopy in Pozniak’s cohort, only 2 of 29 biopsies showed KS [8]. Yet, despite these commonly reported findings and nonspecific bilateral hazy nodular opacities on his chest x-ray (Fig. 2), our patient did not have any characteristic lesions on exam or bronchoscopy. The entire examined tracheobronchial tree was free of lesions and, despite multiple biopsies having been taken, the patient did not have any significant bleeding.

Although our patient did not present with classic skin or mucosal lesions, he did have lymphopenia which is commonly associated with the KS [11, 12]. In the emergency department, his white blood cell count was measured as normal at 4.7 cells/nL (normal range 3.9–10.6 cells/nL) and had a slight neutrophil predominance of 69.4% (normal range 50–60%) but did also demonstrate lymphopenia with an automated count of 16.5% (normal range 25–40%). Records from his prior limited evaluation were unavailable for review or comparison to determine the duration of this finding. Reduced leukocyte and lymphocyte counts are associated with advanced stages of KS [12], but case reports of patients with KS who did not demonstrate typical immunologic deficiencies have also been made [13].

As a 44 year old man in America, our patient should have undergone routine screening for HIV which may have prevented delay in his diagnosis. In 2013, the CDC estimated that 1.2 million persons in the US are living with HIV and approximately 13%, or 1 in 8 individuals, didn’t know they had HIV. CDC guidelines advocate for routine HIV screening as a normal part of general medical practice for all patients aged 13–64 years unless they opt out of testing. The CDC also reports that voluntary HIV screening is cost-effective even in settings where HIV prevalence is low. In fact, the CDC advocates for routine HIV testing to reduce the stigmas associated with testing due to risk behaviors; many people do not perceive themselves at risk or do not disclose their risky behaviors to clinicians [14]. This patient had denied risk factors multiple times and it was not until the KS diagnosis was confirmed and the HIV test was positive that he was willing to discuss high risk sexual behaviors which had reportedly occurred more than 10 years prior. Identification of HIV infection and AIDS-defining KS disease is meaningful for affected patients since the 5-year survival rate has improved from an estimated 10% in the pre-HAART era to approximately 72% with treatment including chemotherapy and HAART [15].

## Conclusion

Pulmonary KS is an uncommon diagnosis, though it presents with common symptoms of dyspnea and cough, occasionally with hemoptysis. Without treatment, patients with pulmonary KS have median survival of months, but with chemotherapy and HAART, they may achieve relief from symptoms and improve survival. Screening guidelines should be followed and diagnostic evaluation pursued for persistently symptomatic patients, even if their symptoms may seem as common as a cough.

## Abbreviations

AIDS: Acquired immunodeficiency syndrome
HHV8: Human herpesvirus 8
HIV: Human immunodeficiency virus
KS: Kaposi’s sarcoma.
